# Vitamin C – a scoping review for Nordic Nutrition Recommendations 2023

**DOI:** 10.29219/fnr.v67.10300

**Published:** 2023-12-28

**Authors:** Jens Lykkesfeldt, Anitra C. Carr

**Affiliations:** 1 Faculty of Health and Medical Sciences, University of Copenhagen, Copenhagen, Denmark; 2 Department of Pathology and Biomedical Science, University of Otago, Christchurch, New Zealand

**Keywords:** vitamin C, ascorbic acid, antioxidants, nutrition recommendations

## Abstract

Vitamin C has multiple metabolic functions in the body, but the available information on the exact relationship between these functions and the intake necessary to maintain them is very limited. However, most attempts to objectively measure adequacy of vitamin C status, including, for example, replacement of metabolic turnover, chronic disease prevention, urinary excretion, and saturation of immune cells and body compartment, currently point toward 50 µmol/L as a reasonable target plasma concentration. As a strong correlation between body weight and vitamin C status exists, recommended intakes (RIs) for other age groups may be extrapolated from the adult RI based on weight. However, as body weights above 70 kg are becoming increasingly common – also in the Nordic region – an RI of 140 mg/day for individuals weighing 100 kg or more should be considered to compensate for the larger volume of distribution. Finally, smoking continues to be a common contributor to poor vitamin C status; therefore, it is proposed that people who smoke increase their daily vitamin C intake by 40 mg/day to compensate for the increased metabolic turnover induced by smoking.

## Popular scientific summary

- Vitamin C (ascorbic acid) is a water-soluble vitamin with multiple roles in the body as an antioxidant and a cofactor for various biochemical processes.
- The major sources of vitamin C in the diet are fresh berries, fruits, and vegetables.
- Plasma concentrations of vitamin C of 50 µmol/L or higher are considered adequate.
- Individual vitamin C status can be influenced by several lifestyle-related and biological factors, such as diet, sex, genetics, body weight, and smoking habits.

Vitamin C is a ubiquitous water-soluble carbohydrate that in spite of its simple and low-molecular-weight structure is essential for human health (1). It is present in most food items in varying amounts with the highest concentrations found in fruits and vegetables, in particular peppers, kiwi and citrus fruits, and many berries (2). In contrast to most living organisms, humans and a few other species have lost the ability to produce vitamin C from glucose, hence making it an essential nutrient. Prolonged and severe vitamin C deficiency leads to the ultimately mortal condition scurvy, and while this may be prevented by ingestion of even small amounts of vitamin C per day (3), the exact dose is being debated (4).

Vitamin C exists in two forms, L-ascorbic acid and L-dehydroascorbic acid, both of which have antiscorbutic properties as most cells readily convert dehydroascorbic acid into the biologically active reduced form, ascorbic acid (5). A vast literature has emerged showing that ascorbic acid is an exceptional biological antioxidant capable of scavenging reactive oxygen and nitrogen species (6), but also that it functions as a specific cofactor for numerous mono-, di-, and mixed-function oxygenases involved in, for example, the formation of connective tissue, synthesis of neurotransmitters, and epigenetic control of gene expression just to mention a few (7, 8).

Besides variation in diet composition, produce quality and preparation, as well as the potential use of fortified food items or supplements, several biological and lifestyle-associated factors are known to influence individual vitamin C status (2, 9). Examples are gender, genotype, body weight, pregnancy, lactation, and smoking habits (10).

The mechanisms governing the absorption, distribution, and excretion of vitamin C are very complex and markedly different from those being used to account for uptake and elimination kinetics of the majority of other small molecules (11). Unfortunately, this has been overlooked in many larger studies in the literature giving rise to misleading interpretations and unwarranted generalizations (12). Consequently, a critical eye is necessary to deduce the information required to propose evidence-based recommendations from the available literature on vitamin C. At the same time, plasma vitamin C status can be considered a biomarker of fruit and vegetable intake, and its potential health benefit may therefore be difficult to isolate from that of a diet rich in these food items (13). The aim of this scoping review is to describe the totality of evidence for the role of vitamin C for health-related outcomes as a basis for setting and updating dietary reference values (DRVs) for the Nordic Nutrition Recommendations 2023 (Box 1).

*Box 1.* Background papers for Nordic Nutrition Recommendations 2023

- This paper is one of many scoping reviews commissioned as part of the Nordic Nutrition Recommendations 2023 (NNR2023) project (14).
- The papers are included in the extended NNR2023 report but, for transparency, these scoping reviews are also published in Food & Nutrition Research.
- The scoping reviews have been peer-reviewed by independent experts in the research field according to the standard procedures of the journal.
- The scoping reviews have also been subjected to public consultations (see report to be published by the NNR2023 project).
- The NNR2023 committee has served as the editorial board.
- While these papers are a main fundament, the NNR2023 committee has the sole responsibility for setting dietary reference values in the NNR2023 project.

## Methods

This review follows the protocol developed within the NNR2023 (14). The sources of evidence used in the scoping review follow the eligibility criteria described previously (15). One qualified systematic review on wholegrains, vegetables, and fruit and the risk of cancer (16), which include an assessment of vitamin C-containing foods, was identified by the NNR2023 project (17). However, this report did not present continuous vitamin C dose-response data and was therefore not considered relevant for the current review. Official reports published by the European Food Safety Association (EFSA) (18, 19) and the National Academy of Sciences, Engineering, and Medicine (NASEM) in the United States (former Institute of Medicine [IOM]) (20) were also consulted.

The literature search for this scoping review was performed on August 01, 2022 in Medline. Due to the overwhelming number of vitamin C-related publications, the literature search was limited to systematic reviews/meta-analyses with the term ‘vitamin C’, or related terminology in the title, using the search string: (vitamin C[Title] OR vitamins C[Title] OR ascorbate[Title] OR ascorbic acid[Title]) AND (2011:3000[pdat]) AND (meta-analysis[Filter] OR systematicreview[Filter]) AND English[Filter]. An additional search was carried out to capture vitamin C-related articles which used the term ‘antioxidant/s’ in the title and ‘vitamin C’ or related terminology in the abstract, using the search string: (antioxidant[Title] OR antioxidants[Title]) AND (vitamin C[Title/Abstract] OR vitamins C[Title/Abstract] OR ascorbate[Title/Abstract] OR ascorbic acid[Title/Abstract]) AND (2011:3000[pdat]) AND (meta-analysis[Filter] OR systematic review[Filter]) AND English[Filter]. A comparable search to this was carried out by substituting the terms ‘micronutrient/s’ for ‘antioxidant/s’. Other reports (e.g. those with meta-analysis in the title, but missed using the meta-analysis filter) were found through additional related searches.

**Fig. 1:**
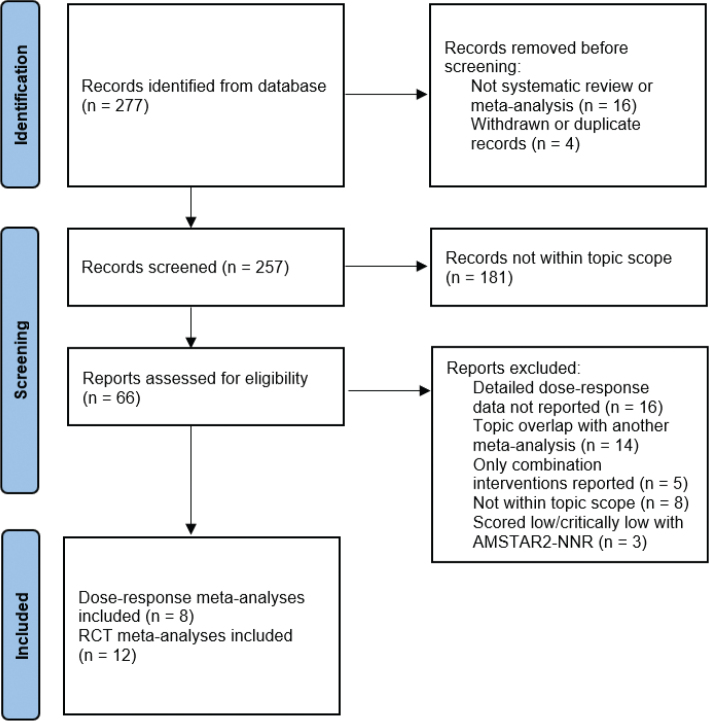
Search strategy to identify relevant meta-analyses for inclusion.

The above search strategies generated a total of 257 systematic reviews/meta-analyses once duplicates and unrelated papers had been removed (Fig. 1). The papers were categorized into topics based on title and keywords: these included cardiovascular health, metabolic health, cancer prevention, immune health, cognitive and mental health, total mortality, and other conditions (e.g. bone health, periodontal health, eye health, and fertility). Categories that were not considered further included combination therapies, use of high-dose intravenous vitamin C, critical care (e.g. sepsis, COVID-19, burns), surgery, postoperative atrial fibrillation, complex regional pain syndrome, exercise outcomes, and treatment of various other diseases. Within each included category, the most up-to-date or the most comprehensive (e.g. dose vs. risk) meta-analyses were selected. Meta-analyses of dose-response observational studies were only included if they provided detailed dose-response data from which estimates of linear departure and maximum effect threshold values for vitamin C intakes and circulating concentrations could be derived. Meta-analyses of randomized controlled trials (RCTs) were only included if they reported vitamin C as the only intervention or vitamin C monotherapy as a subgroup. Publication quality was assessed using the modified AMSTAR2-NNR, a critical appraisal tool for systematic reviews that include randomized or non-randomized studies and/or observational studies (14, 21). Meta-analyses that scored low or critically low using AMSTAR2-NNR were excluded. Note that AMSTAR2-NNR assess the quality of the published meta-analysis, not the quality of the included studies.

The selection process resulted in 8 dose-response meta-analyses of observational studies (Table 1) and 12 meta-analyses of RCTs (Table 2) covering cardiovascular health, blood pressure, cardiometabolic risk factors, cancer prevention, immune health, neurological and mental health, and total mortality. The RCTs included in the selected meta-analyses primarily comprised vitamin C doses >200 mg/day. As such, these meta-analyses were not able to directly contribute to health-related adjustments to the DRVs, but have provided supportive evidence of vitamin C’s health effects. Combining detailed dose versus concentration data with supportive evidence from the dose/concentration versus risk meta-analyses have led to the suggested change in the DRVs as detailed below.

**Table 1:** Summary of dose-response meta-analyses of observational studies.

Reference	Health condition	No./type of studies	No. of participants	Associations of vitamin C with outcomes	Linear dose-response threshold^a^	Maximum dose-response threshold^a^	AMSTAR2-NNR quality score^b^
	Cardiovascular health						
Aune et al. (2018) (22)	CHD risk	12 dietary intakes	241,579	↓ CHD highest versus lowest intakes RR 0.83 (95% CI 0.71, 0.98) 100 mg/day increment RR 0.88 (95% CI 0.79, 0.98)	~85 mg/day	~175 mg/day	Moderate
		4 circulating levels	6,992	↓ CHD highest versus lowest levels RR 0.71 (95% CI 0.59, 0.86) 50 μmol/L increase RR 0.74 (95% CI 0.65, 0.83)	~60 μmol/L	~95 μmol/L	
	Stroke risk	13 dietary intakes	298,066	↓ stroke highest versus lowest intakes RR 0.84 (95% CI 0.77, 0.91) 100 mg/day increment RR 0.92 (95% CI 0.87, 0.98)	~110 mg/day	~200 mg/day	
		5 circulating levels	27,843	↓ stroke highest versus lowest levels RR 0.60 (95% CI 0.49, 0.73) 50 μmol/L increase RR 0.70 (95% CI 0.61, 0.81)	~40 μmol/L	~75 μmol/L	
	CVD risk	11 dietary intakes	246,711	↓ CVD highest versus lowest intakes RR 0.84 (95% CI 0.77, 0.91) 100 mg/day increment RR 0.89 (95% CI 0.85, 0.94)	~90 mg/day	~440 mg/day	
		6 circulating levels	45,273	↓ CVD highest versus lowest levels RR 0.61 (95% CI 0.45, 0.83) 50 μmol/L increase RR 0.76 (95% CI 0.65, 0.87)	ND	~85 μmol/L	
Jayedi et al. (2019) (23)	CVD mortality	10 dietary intakes	242,677	↓ mortality highest versus lowest intakes RR 0·79 (95 % CI 0·68, 0·89) 50 mg/day increment RR 0·92 (95 % CI 0·88, 0·96)	~105 mg/day	~185 mg/day	High
		6 circulating levels	45,040	↓ mortality highest versus lowest levels RR 0·60 (95 % CI 0·42, 0·78) 20 µmol/L increment RR 0·87 (95 % CI 0·80, 0·94)	~35 μmol/L	~60 μmol/L	
	Cancer risk						
Aune et al. (2018) (22)	Total cancer risk	9 dietary intakes	181,318	↓ cancer highest versus lowest intakes RR 0·87 (95 % CI 0·78, 0·95) 100 mg/day increment RR 0.93 (95% CI 0.87, 0.99)	~110 mg/day	~170 mg/day	Moderate
		6 circulating levels	47,678	↓ cancer highest versus lowest levels RR 0·68 (95 % CI 0·57, 0·80) 50 µmol/L increment RR 0·74 (95 % CI 0·66, 0·82)	~40 μmol/L	~95 µmol/L	
Bo et al. (2015) (24)	Esophageal cancer risk	20 dietary intakes	11,018	↓ cancer highest versus lowest OR 0.58 (95% CI 0.49, 0.68) 50 mg/day increment OR 0.87 (95% CI 0.80, 0.93	~80 mg/day	~380 mg/day	Moderate
Li et al. (2014) (25)	Gastric cancer risk	32 dietary intakes	733,894	↓ cancer higher intakes OR 0.58 (95% CI 0.51, 0.65) 100 mg/day increment OR 0.78 (95% CI 0.67, 0.90)	~110 mg/day	~360 mg/day	Moderate
Cao et al. (2016) (26)	Cervical cancer risk	12 dietary intakes	8,831	↓ cancer higher intakes OR 0.58 (95% CI 0.44, 0.75) 50 mg/day increment OR 0.92 (95% CI 0.89, 0.94)	~185 mg/day	~375 mg/day	Moderate
Bai et al. (2015) (27)	Prostate cancer risk	18 dietary intakes	103,658	↓ cancer highest versus lowest intakes RR 0.89 (95%CI: 0.83, 0.94) 150 mg/day increment RR 0.91 (95% CI 0.84, 0.98)	~110 mg/day	~290 mg/day	Moderate
	Neurological health						
Talebi et al. (2022) (28)	Parkinson disease risk	12 dietary intakes	318,784	X PD highest versus lowest intakes RR 0.95 (95% CI 0.77, 1.18) ↓ PD in female participants RR 0.77 (0.62, 0.95) 50 mg/day increment RR 0.94 (95% CI 0.88, 0.99)	ND	~265 mg/day	High
	Mortality						
Jayedi et al. (2018) (29)	All-cause mortality	15 dietary intakes	315,534	↓ mortality highest versus lowest intakes RR 0·88 (95 % CI 0·83, 0·94) 50 mg/day increment RR 0·96 (95 % CI 0·93, 0·98)	~80 mg/day	~130 mg/day	Moderate
		7 circulating levels	45,868	↓ mortality highest versus lowest levels RR 0·61 (95 % CI 0·53, 0·69) 20 µmol/L increment ↓ RR 0·87 (95 % CI 0·83, 0·90)	~35 µmol/L	~95 µmol/L	
Aune et al. (2018) (22)	All-cause mortality	16 dietary intakes	315,214	↓ mortality highest versus lowest intakes RR 0·86 (95 % CI 0·80, 0·92) 100 mg/day increment ↓ 0.89 (95% CI 0.85, 0.94)	~100 mg/day	~185 mg/day	Moderate
		8 circulating levels	47,238	↓ mortality highest versus lowest levels RR 0·68 (95 % CI 0·60, 0·77) 50 µmol/L increment ↓ RR 0·72 (95 % CI 0·66, 0·79)	ND	~95 µmol/L	

a Dose- and concentration-dependence thresholds were estimated from linear departure and maximum effect points on dose-response curves. Mean linear intake threshold ~105 mg/day (median ~105 mg/day); mean maximum intake threshold ~260 mg/day (median ~235 mg/day); mean linear concentration threshold ~40 µmol/L (median ~40 µmol/L); mean maximum concentration threshold ~85 µmol/L (median ~95 µmol/L).

b AMSTAR2-NNR assesses the quality of the published meta-analysis, not the quality of the included studies. CHD, coronary heart disease; CI, confidence interval; CVD, cardiovascular disease; ND, not determined; OR, odds ratio; PD, Parkinson disease; RR, relative risk.

**Table 2:** Summary of meta-analyses comprising randomized controlled trials.

Reference	Health condition	No./type of studies	No./type of participants	Vitamin C doses	Vitamin C duration	Effect of vitamin C intervention	AMSTAR2-NNR quality score^a^
	Cardiovascular health						
Al-Khudairy et al. (2017) (30)	CVD prevention	1 RCT	14,641 male physicians	0.5 g/day	8 years	X CVD events HR 0.99 (95% CI 0.89 to 1.10)	High
Ashor et al. (2014) (31)	Endothelial function	44 RCT	1,129 various	0.09–3 g/day	1–56 days	↑ endothelial function (SMD: 0.50, 95% CI 0.34, 0.66; *P* < 0.001) Effects larger if atherosclerotic (SMD: 0.84, 95% CI 0.41, 1.26) Dose-dependent improvement in EF (>500 vs. ≤500 mg/day)	Moderate
	Blood pressure						
Mason et al. (2021) (32)	Blood pressure	8 RCT	466 T2DM	0.5–1.5 g/day	3–17 weeks	↓ SBP MD −6.27 (95% CI −9.60, −2.96) mmHg; *P* = 0.0002 ↓ DBP MD −3.77 (95% CI −6.13, −1.42) mmHg; *P* = 0.002	Moderate
Guan et al. (2020) (33)	Blood pressure	8 RCT	614 hypertensive	0.3–1 g/day	4–24 weeks	↓ SBP WMD −4.09 (95% CI −5.56, −2.62) mmHg; *P* < 0.001 ↓ DBP WMD −2.30 (95% CI −4.27, −3.31) mmHg; *P* = 0.02	Moderate
Juraschek et al. (2012) (34)	Blood pressure	15 RCT (monotherapy)	703 various	0.3–4 g/day	3–12 weeks	↓ SBP effect size −2.59 (95% CI: −3.81, −1.38) mmHg X DBP effect size −0.52 (95% CI: −2.07, 1.04) mmHg Effects tended to be larger in hypertension and diabetes	Moderate
	Cardiometabolic risk factors						
Mason et al. (2021) (32)	Glycaemic control	28 RCT	1,574 T2DM	0.2–3 g/day	2–52 weeks	↓ FG MD −0.74 (95% CI −1.17, −0.31) mmol/L; *P* = 0.0007 ↓ HbA1c MD −0.54% (95% CI −0.90, −0.17); *P* = 0.004	Moderate
	Lipid levels					↓ TG MD −0.20 (95% CI −0.36, −0.04) mmol/L; *P* = 0.01 ↓ TC MD −0.27 (95% CI −0.43, −0.10) mmol/L; *P* = 0.001 X LDL-C MD −0.23 (95% CI −0.48, 0.03) mmol/L; *P* = 0.08 X HDL-C MD 0.06 (95% CI 0.00, 0.13) mmol/L; *P* = 0.06	
Khodaeian et al. (2015) (35)	Insulin resistance	3 RCT	92 T2DM	0.8–1 g/day	4–16 weeks	X HOMA SMD −0.150 (95% CI −0.494, 0.194); *P* = 0.39	Moderate
Ashor et al. (2017) (36)	Glycaemic control	22 RCT	937 various	0.07–6 g/day	1–120 days	X glucose, HbA1c, insulin overall â glucose in T2DM (−0.44 mmol/l, 95% CI −0.81, −0.07, *P* = 0.01) Effects on glucose greater with higher baseline glucose and BMI	Moderate
Ashor et al. (2016) (37)	Lipid levels	40 RCT	1,981 various	0.125–4.5 g/day	2–240 weeks	X lipid levels overall ↓ TC younger participants (−0.26 mmol/L, 95% CI −0.45, −0.07) ↓ LDL-C healthy participants (−0.32 mmol/L, 95% CI −0.57, −0.07) ↓ TGs T2DM (−0.15 mmol/L, 95% CI −0.30, −0.002) ↑ HDL-C T2DM (0.06 mmol/L, 95% CI 0.02, 0.11) Effects on TC and TG greater with higher baseline lipid levels Effects on HDL-C greater with lower baseline vitC levels	Moderate
Jafarnejad et al. (2018) (38)	C-reactive protein levels	12 RCT	893 various	0.2–3 g/day	21–365 days	↓ CRP −0.23 mg/L (95% CI −0.44, -0.03), *P* = 0.02 Effects larger with baseline CRP ≥ 3 −1.48 mg/L (95% CI −2.84, −0.11)	High
	Cancer prevention						
Lee et al. (2015) (39)	Cancer incidence	2 RCT (monotherapy)	22,268	0.5 g/day	8–9.4 years	X cancer RR 1.03 (95% CI 0.95, 1.10)	Moderate
	Immune health						
Abioye et al. (2021) (40)	Common cold risk	24 RCT	10,961 adults	< 0.25–2 g/day	2 weeks >1 year	↓ common cold RR 0.96 (95% CI 0.93, 0.99); *P* = 0.01 ↓ common cold males RR 0.82 (95% CI 0.70, 0.96) ↓ common cold MIC RR 0.65 (95% CI 0.47, 0.89)	Moderate
	Acute respiratory infection duration	24 RCT	8,344 adults			↓ ARI duration by −9% (95% CI −16, −2); *P* = 0.014 ↓ common cold duration −9% (95% CI −16, −3); *P* = 0.007	
	Mental health						
Yosaee et al. (2021) (41)	Depression	10 RCT	836 various	0.1–2 g/day	2–24 weeks	X mood status effect size 0.09 (95% CI −0.15, 0.33); *P* = 0.47 ↓ subclinical depression effect size −0.18 (95% CI -0.35, -0.01); *P* = 0.04	High

a AMSTAR2-NNR assesses the quality of the published meta-analysis, not the quality of the included studies. Abbreviations: ARI, acute respiratory infection; CRP, C-reactive protein; CVD, cardiovascular disease; CI, confidence interval; DBP, diastolic blood pressure; EF, endothelial function; FG, fasting glucose; HbA1c, glycated hemoglobin; HDL-C, high-density lipoprotein cholesterol; HOMA, homeostasis model assessment; IV, intravenous; LDL-C, low-density lipoprotein cholesterol; LVEF, left ventricular ejection fraction; MD, mean difference; MIC, middle-income countries; RCT, randomized controlled trial; RR, relative risk; SBP, systolic blood pressure; SMD, standardized mean difference; TC, total cholesterol; TG, triglycerides;T2DM, type 2 diabetes mellitus.

## Physiology

Much of the present knowledge on vitamin C functions and behavior under physiological conditions has been realized through laboratory and experimental animal studies. In particular, useful experimental models have included isolated human cells (42–46), the gulonolactone oxidase (GULO) knockout mouse, genetically modified not to produce vitamin C (47), and the guinea pig, as this species is among the very few that, like humans, naturally lacks the ability to biosynthesize ascorbic acid (48–50).

L-Ascorbic acid is a low-molecular-weight electron donor that has the capacity to reduce any biologically relevant oxidant species as well as regenerate other antioxidants, such as vitamin E, from their oxidized forms (6, 51). It is normally present in relatively large concentrations and provides antioxidant protection in body fluids such as plasma, semen, and cerebrospinal fluid, where antioxidant enzymes are not present, as well as in several immune and various other cell types (52). Moreover, ascorbic acid provides reducing equivalents improving non-haem iron uptake and is necessary for maximal activity of many oxygenase-type enzymes containing metal-ion catalytic sites involved in a wide variety of metabolic pathways including, for example, mature triple-helix collagen formation, catecholamine neurotransmission, mitochondrial function, cholesterol elimination, endothelial function, peptide hormone function, and epigenetic regulation (7, 8).

**Fig. 2:**
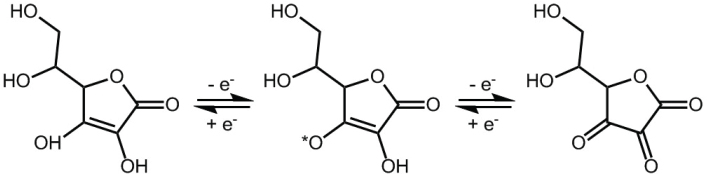
Chemical structures of ascorbic acid, ascorbyl free radical, and dehydroascorbic acid.

Due to its low pKa value of 4.2, the ionized form, ascorbate, is the predominant form constituting >99.9% at physiological pH. Providing electrons either as antioxidant or enzyme cofactor oxidizes ascorbate to dehydroascorbic acid; however, the oxidized form is rapidly transported into cells through concentration gradient-driven glucose transporters (GLUT) and efficiently reduced back to ascorbate intracellularly and thus ‘recycled’ by most cell types in the body (53) (Fig. 2).

The absorption, distribution, metabolism, and excretion of vitamin C differ substantially from that of most other low-molecular-weight compounds in that it is highly dose-dependent, tissue-specific, and regulated by vitamin C status *per se* (11). Transport of vitamin C across membranes is governed by active and saturable sodium-dependent vitamin C co-transporters (SVCTs) that are energy-dependent membrane-spanning enzymes responsible for building up the considerable vitamin C concentration gradients observed between body compartments (54). Thus, some organs such as the brain have vitamin C concentrations up to 10 mM superseding that of other important cellular antioxidants including glutathione (50, 55). SVCT forms and expression differ between tissues resulting in a highly diverse distribution pattern with specific tissues getting priority over others (56, 57). This is particularly observed during periods of deficiency, where selective retention of vitamin C occurs in, for example, the brain and adrenal glands, which indicates important functions for the vitamin in these organs, whereas other tissues such as liver are depleted as rapidly as plasma (48, 50, 58).

When the body experiences an inadequate intake of vitamin C, selective mechanisms for retaining ascorbate are activated. The kidneys have an inducible SVCT in the proximal tubules capable of efficiently reabsorbing ascorbate from the urine (54). This dynamic process limits the loss of vitamin C through the urine to almost zero during deficiency, while reabsorption is completely shut down during conditions of high vitamin C intake, thereby efficiently excreting surplus amounts (59). Also, the intestinal SVCT has the ability to vary vitamin C uptake with availability, thus helping to keep the vitamin C status of the body within a relatively narrow homeostatic range (60). As absorption, distribution, and excretion of vitamin C are tightly controlled by the active SVCTs, this opens the possibility of genetic polymorphisms playing a role in vitamin C homeostasis between subpopulations. Indeed, several genetic variants have been identified that appear to result in an either higher or lower homeostatic set point (61). However, the dose versus concentration data accumulated so far do not allow for specific conclusions with regard to the potential health perspectives of having the various genotypes.

## Vitamin C pharmacokinetics in healthy people

**Fig. 3:**
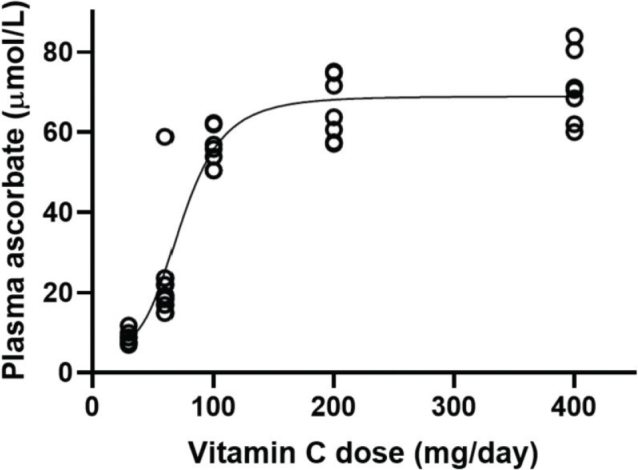
Plasma ascorbate concentrations in healthy volunteers as a function of daily dose. Figure from (65); Creative Commons Attribution (CC BY) license (https://creativecommons.org/licenses/by/4.0/).

Collectively, the above mechanisms result in a maximal achievable steady-state fasting plasma vitamin C concentration of approximately 70–80 µmol/L in healthy people (Fig. 3). Supplementation with multiple daily supraphysiological doses may transiently drive up the plasma concentration, but it will quickly revert once multiple daily dosing is stopped (62). Pharmacokinetic studies have revealed that in healthy young men and women, doses less than about 60 mg/day are quantitatively absorbed (63). From 60 mg/day, some vitamin C excretion is observed and it gradually increases with increasing doses. The steady-state plasma concentration continues to increase to the maximal 70–80 µmol/L reached at intakes of about 400–500 mg/day in healthy individuals (63). At higher supraphysiological doses, excess vitamin C is quantitatively excreted through the urine with a half-life of about 2 h (64).

## Impact of body weight

Body weight and volume appear to be determining factors for the dose versus concentration relationship of vitamin C (65, 66). The difference observed between men and women regarding their plasma status resulting from a comparable dietary intake seems to result primarily from differences in size and body composition rather than gender *per se* (67). Moreover, an inverse correlation between body weight and vitamin C status has been observed in both men and women. Likewise, pregnancy is also associated with a declining vitamin C status of the mother (10). Interestingly, this decline may also be partly explained by the increasing body weight occurring during pregnancy *per se* rather than solely by a preferential allocation of vitamin C to the fetus.

**Fig. 4:**
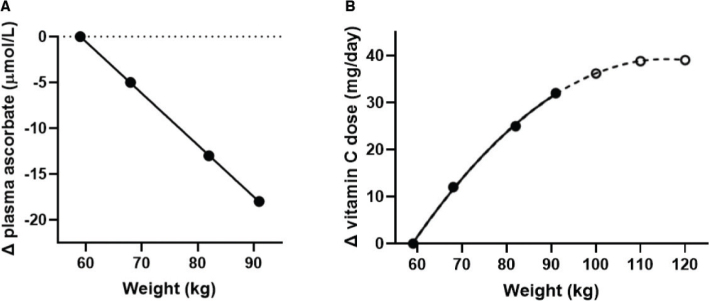
Decreasing plasma ascorbate concentrations with increasing body weight (A) and increasing vitamin C requirements with increasing body weight (B). The dashed line represents extrapolation of the weight data points. Figure from (65); Creative Commons Attribution (CC BY) license (https://creativecommons.org/licenses/by/4.0/).

**Fig. 5:**
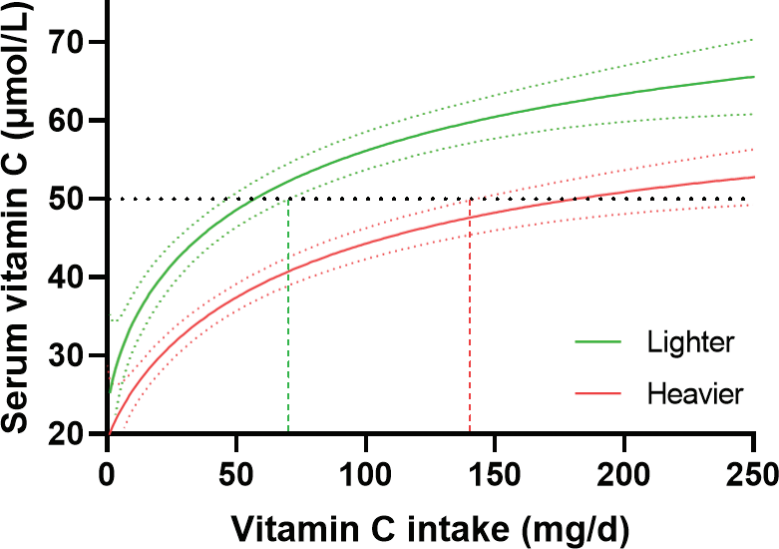
Analysis of NHANES 2017/2018 data indicated a twofold higher requirement for vitamin C for the heavier tertile (*n* = 930) relative to the lighter tertile (*n* = 932). Sigmoidal (four-parameter logistic) curves with asymmetrical 95% confidence intervals were fitted to dose-concentration data to estimate the vitamin C intakes required to reach ‘adequate’ serum vitamin C concentrations of 50 µmol/L (dashed line). Figure from (74); Creative Commons Attribution (CC BY) license (https://creativecommons.org/licenses/by/4.0/).

In line with global trends, the prevalence of obesity and overweight continues to increase in the Nordic and Baltic countries, with the prevalence of obesity ranging from 15 to 30% and the prevalence of overweight ranging from 24 to 53% for adult females and males (68). Large epidemiological studies, including the US NHANES (69), the Canadian Health Measures Survey (70), the UK EPIC-Norfolk study (71), and the French SU.VI.MAX baseline (72), have all indicated inverse correlations between body weight or body mass index (BMI) and vitamin C status, supporting a volumetric dilution effect (67). This premise has been further supported by an intervention study by Block et al. (66), in which attenuated vitamin C status was observed in overweight and obese people, despite comparable dietary intake to people of normal weight. In response, the authors proposed that vitamin C recommendations should be based on a ‘dose per kg body weight’ or in terms of ‘desirable plasma concentrations’. Recent reanalysis of the Block study data (66), in combination with the Levine pharmacokinetic study data (59), suggests that an additional 10 mg/day of vitamin C may be required for every additional 10 kg of body weight within the range of 60–90 kg (Fig. 4) (65). Obesity may increase vitamin C requirements further due to elevated inflammation and oxidative stress (73). In fact, analysis of ‘real-world’ NHANES data indicated that people in the heavier tertile had a twofold higher requirement for vitamin C than those in the lighter tertile to reach adequate circulating concentrations of the vitamin (Fig. 5) (74). This corresponded to >20 mg/day of vitamin C required for every additional 10 kg in weight gain.

## Impact of smoking

**Fig. 6:**
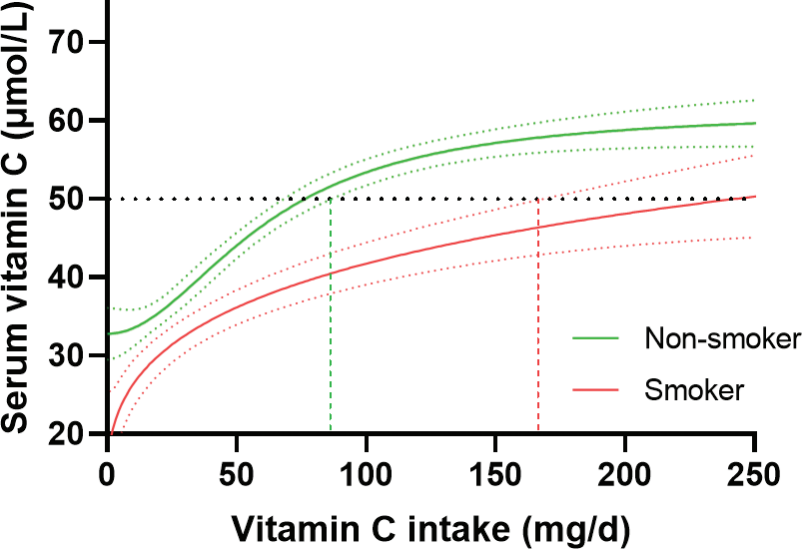
Analysis of NHANES 2017/2018 data indicated a twofold higher requirement for vitamin C for smokers (*n* = 681) relative to non-smokers (*n* = 2,068). Sigmoidal (four-parameter logistic) curves with asymmetrical 95% confidence intervals were fitted to dose-concentration data to estimate the vitamin C intakes required to reach ‘adequate’ serum vitamin C concentrations of 50 µmol/L (dashed line). Figure from (74); Creative Commons Attribution (CC BY) license (https://creativecommons.org/licenses/by/4.0/).

Smoking continues to be relatively common in the Nordic and Baltic countries with a prevalence ranging from 10 to 44% for adult females and males (75). Both active and passive smoking are known to increase oxidative stress and enhance the utilization of vitamin C (76–81). As such, smokers have higher requirements for the vitamin than non-smokers due to a higher metabolic loss (11, 82, 83). In support of this, numerous observational studies (including NHANES, the Canadian Health Measures Survey, EPIC-Norfolk, and the French POLA study and SU.VI.MAX baseline) have reported lower vitamin C status and a higher prevalence of deficiency in smokers relative to non-smokers (70, 72, 84–86). In addition, smokers generally have a lower dietary intake of vitamin C, further contributing to a poor vitamin C status (79, 87). Some authorities have considered these factors with higher vitamin C recommendations for smokers comprising additional intakes of 20–80 mg/day over the DRV for adults in these countries (10). However, it is likely that smokers need substantially more vitamin C than these recommendations to fully compensate for the effect of smoking on plasma vitamin C status (79, 82). In confirmation of this premise, analysis of NHANES 2017/2018 data indicated that smokers had a twofold higher requirement for vitamin C than non-smokers to reach adequate circulating concentrations of the vitamin (Fig 6) (74).

## Deficiency

Plasma vitamin C deficiency (concentrations ≤11 µmol/L) indicates that tissues will also be depleted as there is a close relationship between plasma and tissue values (88). Deficiency affects the pharmacokinetic profile of plasma vitamin C in that, following supplementation, plasma vitamin C values will not increase until the tissues are no longer depleted, due to preferential uptake of the vitamin into depleted tissues (11, 48, 50). Vitamin C deficiency is relatively uncommon in European countries (89). Two population studies on vitamin C status have been carried out in Finland: one in North Karelia (1992–2002) in >1,600 adults aged 25–64 years (90, 91) and one in Eastern Finland (1984–1989) in >1,600 men aged 42–65 years (92). The mean vitamin C status for men ranged from 37 to 48 µmol/L, with a prevalence of deficiency of 2.2–5.7% (90–92). Vitamin C deficiency is a risk factor for clinical scurvy, which, although rare, is still observed in individuals in high-income countries.

### Toxicity

Vitamin C, being a small water-soluble molecule, is readily filtered by the kidneys; therefore, any excess not required by the body is readily excreted in urine (11). Thus, vitamin C has no known upper limit (UL) for toxicity, although some authorities have set ULs of 1–2 g/day (10). Adverse side effects are mostly related to gastrointestinal disturbance due to unabsorbed vitamin C from high gram doses passing through the gastrointestinal tract (93). The evidence for a role of oral vitamin C in kidney stone formation is currently poor and contradictory (93).

## Assessment of nutrient status

Vitamin C intakes correlate with plasma ascorbate concentrations to a certain extent. However, intake is not an ideal proxy for in vivo ascorbate status for a number of reasons, including inherent inaccuracies in intake assessments and the non-linear nature of the dose-concentration relationship (12, 59). Furthermore, numerous factors can influence vitamin C status irrespective of dietary intake (2). As such, the most commonly used marker of vitamin C status is plasma ascorbate concentrations. As vitamin C is not protein bound, free ascorbate can be readily measured in plasma that has been acidified to remove protein, with the preferred assessment methodology being high-performance liquid chromatography with electrochemical detection (94). As increased urinary ascorbate excretion occurs when plasma concentrations have reached the urinary reabsorption threshold (59), urinary ascorbate can potentially be used as a proxy for ‘adequate’ plasma concentration.

Ascorbate is actively accumulated by circulating leukocytes in a dose-dependent manner up to doses of ~100 mg/day (59, 95). Leukocyte ascorbate content correlates with plasma concentrations up to saturating concentrations (88). As such, these cells are often used as a proxy for body tissue ascorbate status due to ease of isolation. Near saturation of neutrophil ascorbate status was used by the IOM to inform their most recent increase in vitamin C recommendations (20). Nevertheless, different tissues accumulate vitamin C to variable extents based on their requirements (96). For example, although muscle biopsy is possible and muscle ascorbate status appears to correlate with intake and plasma concentrations (88), this is an invasive procedure and muscle ascorbate concentrations are relatively low compared with other tissues with higher vitamin C requirements, such as the adrenal and pituitary glands (96). It is noteworthy that the Km of ascorbate-dependent dioxygenases (i.e. the concentration of ascorbate that permits the enzyme to achieve half Vmax) ranges between 180 and 300 µmol/L (97, 98), which is indicative of intracellular requirements.

To date, there is no definitive biomarker for vitamin C functional requirements. In 1991, Levine and co-workers (99) explored the possibility of determining vitamin C requirements via assessing the in vitro conversion of tyrosine to norepinephrine, which comprises vitamin C-dependent steps. In these experiments, adrenal medulla chromaffin cells in culture were incubated with increasing concentrations of ascorbate, and a dose-dependent increase in norepinephrine generation was observed up to 1 mmol/L ascorbate. The extracellular scavenging of neutrophil-derived superoxide radicals by increasing concentrations of ascorbate was used by the IOM as evidence of vitamin C’s antioxidant scavenging effects (20). A dose-dependent scavenging effect was observed for concentrations of up to ~120 µmol/L ascorbate with neutrophils (1 × 10^6^/mL) activated in vitro (100). However, it has not been possible to translate these in vitro findings into in vivo vitamin C requirements.

The use of leukocytes as surrogates to assess vitamin C-dependent epigenetic modifications may in future provide useful information around dietary requirements (10). In preliminary studies, positive correlations were observed between vitamin C status and vitamin C-dependent epigenetic marks in leukocyte DNA, whereby participants with plasma vitamin C concentrations >40 µmol/L exhibited higher concentrations of these epigenetic marks than those with plasma concentrations <20 µmol/L (101). As such, more research in this area appears warranted. Vitamin C’s well-known cofactor role in collagen cross-linking has also been proposed as a potential functional test for adequacy of vitamin C status (102). In this research, urinary excretion of specific cross-link ratios was higher in children with higher vitamin C intakes. A supplementation study (100 mg/day of vitamin C for 7 weeks in children with low baseline intakes) did not, however, alter the cross-link ratio. This may have been due to the supplementation period being insufficient as collagen can have a long turnover in some tissues.

In the absence of definitive functional assays for vitamin C requirement, and the difficulty in obtaining tissue ascorbate concentrations, plasma ascorbate concentrations are currently used to define sufficiency. At present, the most widely accepted plasma ascorbate thresholds are ≤11 µmol/L for deficiency, ≤23 µmol/L for hypovitaminosis C, ≥50 µmol/L for adequate, and ≥70 µmol/L for saturating status. A plasma ascorbate concentration of 50 µmol/L equates to an intake of approximately 100 mg/day (59); this has been used by the EFSA and DACH to help establish their dietary recommendations for vitamin C (18, 103).

## Dietary intake in Nordic and Baltic Countries

The major sources of vitamin C in the diet are fresh fruit and vegetables, specifically guava, kiwifruit, citrus, strawberries, chili pepper, kale, and other brassica (2). Potatoes have a relatively low content of vitamin C; however, due to the generally large quantities consumed, these can be an important source of the vitamin (104). Additionally, the vitamin C content of fresh fruit and vegetables can vary by season and careful food preparation is required to avoid further loss of the vitamin (105, 106). Low dietary intake of fruit and vegetables will have a detrimental effect on vitamin C status, which has been observed in people on restricted diets. It should also be noted that vitamin C content can vary dramatically between different fruit and vegetables (2); therefore, consumption of a variety of fruit and vegetables is encouraged. Poor dietary sources of vitamin C include grains, legumes, nuts, seeds and animal products; meat (other than liver), eggs, and milk (2). According to Nordic and Baltic national dietary surveys, the average dietary intake of vitamin C is in the range of 93–115 mg/day in the Nordic countries and 72–132 mg/day in the Baltic countries (107).

## Health outcomes of relevance to Nordic and Baltic countries

### Cardiovascular health

Two recent dose-response meta-analyses of observational studies have indicated that higher vitamin C intakes (10–13 studies) and circulating concentrations (4–6 studies) are associated with a lower risk of coronary heart disease, stroke, and cardiovascular disease (22) and a lower risk of cardiovascular disease mortality (23). Dose-response and concentration-dependence thresholds were estimated from the linear deviation and maximum effect points on dose-response curves (Table 1). Doses of ~85–110 mg/day were associated with linear risk reduction of these conditions and doses of ~175–200 mg/day were associated with maximal reduction in risk for coronary heart disease, stroke, and cardiovascular disease mortality (with up to ~440 mg/day being associated with increased risk reduction for cardiovascular disease). Concentration-dependence curves indicated that plasma vitamin C concentrations of ~35–60 µmol/L were associated with linear risk reduction and concentrations of ~60–95 µmol/L (i.e. saturating circulating concentrations) were associated with maximal risk reduction for these conditions. Of note, circulating concentrations were more strongly correlated with risk reduction than dietary intakes (22, 23).

A Cochrane meta-analysis of RCTs for the primary prevention of cardiovascular disease identified only one eligible study (the Physicians’ Health Study II), comprising >14,600 participants supplemented with 500 mg/day vitamin C for up to 8 years, in which no effect of supplementation was seen on cardiovascular disease events (Table 2) (30), noting the limitations of vitamin C supplementation RCTs discussed below. An earlier meta-analysis assessing 44 RCTs of vitamin C intervention (doses ≥90 mg/day) on endothelial function reported dose-dependent enhancement in endothelial function, with effects being larger for those with atherosclerosis (31).

### Blood pressure

No dose-response meta-analyses assessing associations between vitamin C intake or circulating concentrations and blood pressure were identified. Three meta-analyses of RCTs that assessed vitamin C supplementation (doses ≥300 or ≥500 mg/day) for reduction of blood pressure were identified; one of these comprised 15 monotherapy RCTs of various cohorts (34), one 8 RCTs of hypertensive participants (33), and the other 8 RCTs of participants with type 2 diabetes (32). Significant decreases in systolic blood pressure were observed in all three meta-analyses, and decreases in diastolic blood pressure in participants with hypertension and diabetes (Table 2). The effect sizes were larger in participants with hypertension and diabetes.

### Cardiometabolic risk factors

Meta-analyses of RCTs have indicated that vitamin C supplementation can improve cardiometabolic risk factor markers (e.g. lipid profiles, glycaemic control, C-reactive protein; Table 2). Two meta-analyses assessed lipid levels following supplementation with vitamin C (doses ≥125 mg/day) in 40 RCTs comprising mixed cohorts (37) and 28 RCTs of people with type 2 diabetes (32). The largest effects were observed in groups with elevated baseline lipids, type 2 diabetes, and lower baseline vitamin C concentrations. Similarly, meta-analyses have assessed the effects of vitamin C intervention (doses ≥70 or ≥200 mg/day) on glycaemic control biomarkers and showed decreased fasting glucose and HbA1c in participants with type 2 diabetes (32, 36). Effects on glucose were greater with higher baseline glucose and higher BMI. However, an earlier meta-analysis of three RCTs did not find an effect of vitamin C supplementation (≥800 mg/day) on reported or estimated insulin resistance in participants with type 2 diabetes (35). A final meta-analysis of 12 RCTs indicated vitamin C supplementation (doses ≥200 mg/day) decreased C-reactive protein concentrations, particularly in those with elevated C-reactive protein at baseline (38).

### Cancer prevention

Dose-response meta-analyses have indicated decreased total cancer risk (22) and decreased risk of cancers at specific sites (e.g. esophageal, gastric, prostate, and cervical) with increasing vitamin C intake or status (24–27). Aune et al. (22) assessed nine dietary intake studies and six studies reporting circulating concentrations which indicated dietary intakes of ~110–170 mg/day and circulating concentrations of ~40–95 µmol/L provided linear to maximum protection, respectively, against total cancer risk (Table 1). The other dose-response meta-analyses indicated that vitamin C intakes of ~80–380 mg/day provided linear to maximal risk reduction, respectively, for cancers at specific sites (24–27). One meta-analysis comprising RCTs assessing vitamin C supplementation for cancer prevention (39) identified two studies (the Physicians’ Health Study II and the Women’s Antioxidant Cardiovascular Study). These, however, showed no effect on total cancer incidence of supplementation with 500 mg/day of vitamin C (Table 2), once again noting the limitations of vitamin C supplementation RCTs discussed below.

### Immune health

No dose-response meta-analyses assessing associations between vitamin C intake or circulating concentrations and infection risk were identified. A recent meta-analysis assessing vitamin C supplementation for acute respiratory tract infections indicated only a small risk reduction in the general population (40) (Table 2). However, enhanced risk reduction was observed in males relative to females and in populations from middle-income countries relative to those from high-income countries (40); both of these subgroups tend to present with lower vitamin C status at baseline (89). Vitamin C supplementation was also shown to reduce the duration of the common cold.

### Neurological and mental health

There is as yet limited evidence from meta-analyses that vitamin C has an effect on neurological and mental health. A dose-response meta-analysis comprising 12 dietary intake studies showed limited association of vitamin C intake with Parkinson disease (28), other than at the highest intake of ~265 mg/day (Table 1). A meta-analysis of 10 RCTs showed no effect of vitamin C supplementation (doses ≥100 mg/day) on mood status in adults (41), apart from a small effect in the group of participants with subclinical depression (i.e. those not prescribed anti-depressants; Table 2).

### Total mortality

Dose-response meta-analyses of up to 16 observational studies have indicated that vitamin C intakes of ~80–185 mg/day are associated with linear to maximal decreases, respectively, in all-cause mortality risk (22, 29). Circulating concentrations of vitamin C (~35–95 µmol/L) from up to eight studies were associated with linear and maximal decreases, respectively, in mortality risk (Table 1). No meta-analyses of RCTs investigating vitamin C as a monotherapy for all-cause mortality were identified.

## Requirement and recommended intakes

**Fig. 7:**
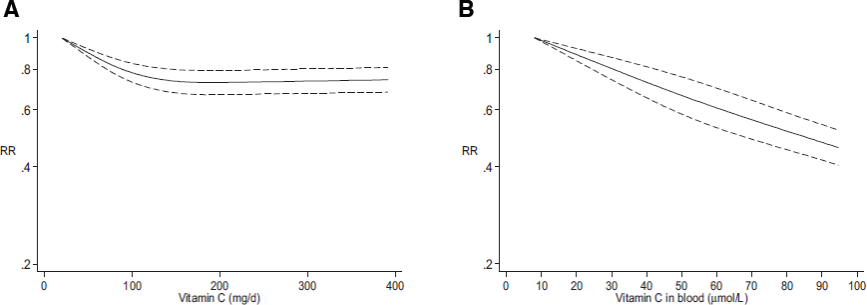
Coronary heart disease risk relative to vitamin C dietary intake (A) and blood concentrations (B): dose-response analyses. Similar trends were observed for stroke and cardiovascular disease. Solid lines represent best-fitting cubic spline and dashed lines 95% CI. Reproduced from (22); Creative Commons Attribution Non-Commercial License (http://creativecommons.org/licenses/by-nc/4.0/).

As outlined earlier, a substantial body of epidemiological studies and associated dose-response meta-analyses have shown an inverse relationship between the plasma concentration of vitamin C and risk of major diseases such as coronary heart disease, stroke, and cancer as well as all-cause mortality. In parallel, a number of the randomized controlled trials in the meta-analyses outline earlier have shown little or no health benefit of supplementation to healthy individuals who already have adequate or saturated vitamin C levels (in contrast to those at risk of vitamin C insufficiency, e.g. hypertensive or diabetic). The essence of these apparently contradictory results is illustrated in Fig. 7 showing that the relative risk of coronary heart disease gradually declines for intakes up to about 175 mg/day (typically obtained mainly by dietary intake among healthy individuals), while the relationship remains unchanged for higher intakes (mostly resulting from concurrent supplementation) (Fig. 7A; [22]). At the same time, an approximately linear relationship is found between increasing plasma concentrations within the physiologically achievable range and decreasing relative risk of coronary heart disease (Fig. 7B). Therefore, besides revealing a stronger correlation between plasma vitamin C status and disease risk than for vitamin C intake, these data may also explain why many intervention studies with vitamin C have little impact in the general population.

Collectively, the meta-analyses suggest that a plasma concentration of about 50 µmol/L is necessary to achieve a mean risk reduction of 30% in morbidity and mortality from chronic diseases (Table 1). The intake necessary to maintain a plasma concentration of 50 µmol/L is also sufficient to replace metabolic turnover of vitamin C as calculated by experiments with radiolabelled vitamin C (108). New evidence suggests that the urinary threshold for vitamin C is approximately 50 µmol/L in healthy individuals (109), thus providing additional support for the target level already regarded as ‘adequate’ by, for example, EFSA (18). Finally, based on pharmacokinetic data, a plasma concentration of at least 50 µmol/L is required to saturate immune cells (95) and muscle tissue (88).

Consequently, a plasma concentration of 50 µmol/L should be chosen as the basis for calculation of the average requirement (AR). Pharmacokinetic studies in men and women suggest that ingestion of approximately 90 mg vitamin C/day will result in a plasma concentration of 50 µmol/L. EFSA reached the same value in their most recent recommendation by calculating the amount necessary to compensate for metabolic losses of 50 mg/day with an estimated uptake efficiency of 80% and a 25% urinary excretion. Thus, with an AR of 90 mg/day and assuming a 10% CV, a recommended intake (RI) of 110 mg/day can be expected to provide 95% of the population with adequate vitamin C concentrations based on a 70 kg body weight.

### Infants

There is insufficient data to set an AR for infants (aged <2 years). Previous recommendations by various health authorities have been based on estimated intakes from human milk or the intake known to prevent scurvy with an ample margin of safety. These recommendations range from 20 to 55 mg/day. Based on these considerations and allowing a conservative safety margin, while considering the very high plasma concentrations seen in newborns that may indicate a higher requirement but at least show that higher steady-state levels are normal in infants, the RI is suggested to be set at 30 mg/day.

### Children and adolescents

There is insufficient data to set an AR for children and adolescents. Previous recommendations have been calculated from the adult AR using isometric scaling based on body weight or by applying estimated growth rates. Here, RIs are obtained by isometric scaling assuming a CV of 10% and are rounded to the nearest 5 mg. Suggested recommendations are 30 mg/day (2–5 years), 45 mg/day (6–9 years), 60 mg/day (10–13 years), and 90 mg/day (14–17 years).

### Adults

The suggested AR for adults is based on the intake necessary for a 70 kg individual to obtain a plasma concentration of about 50 µmol/L, that is, an AR of 90 mg/day. Assuming a CV of 10% results in an RI of 110 mg/day for both men and women with a bodyweight of 70 kg. As discussed earlier in the section on the impact of body weight, there is considerable evidence suggesting that women achieve slightly higher plasma concentrations compared to men from the same intake (89), but also that this discrepancy is mainly due to difference in volume of distribution and can be accounted for by correcting for difference in weight (67). Indeed, the authorities that have implemented different recommendations for men and women have all derived their recommendations for women from that for men based on the weight difference (10). Calculations based on controlled dose versus concentration studies as well as steady-state pharmacokinetics (65) suggest that 10 mg/day per 10 kg body weight should be added/subtracted to obtain the vitamin C necessary to achieve the target plasma concentration of 50 µmol/L with a probability of 95% within the range of a normal body mass calculated from a 70 kg/110 mg/day starting point.

### Elderly

There are insufficient data to set an AR for elderly. Studies have produced mixed results trying to determine if aging *per se* is associated with lower vitamin C status, lower intake of vitamin C or a changed relationship due to altered volume of distribution.

### Pregnancy

There is insufficient data to set an AR for pregnant women. However, consistent evidence suggests that plasma vitamin C decreases gradually throughout pregnancy presumably due to selective uptake by the fetus and the increased total volume of distribution. Most authorities have arbitrarily estimated that an additional intake of 10 mg/day is warranted, and this is consistent with the weight-based approach mentioned earlier. Thus, suggested additional recommended vitamin C intake during pregnancy is +10 mg/day.

### Lactation

Lactation actively removes vitamin C from the mother. Mean concentrations of vitamin C in milk range from 35 to 90 mg/L and an average of 40 mg/day has been estimated to be excreted through milk. Assuming an absorption efficiency of 80% and a CV of 10%, this results in suggested additional RI of +60 mg/day for breastfeeding women.

### Large or overweight individuals

While the inverse relationship between body weight and vitamin C plasma concentration is well established and used actively in the estimation of RIs for women and children (10), health authorities have so far not derived specific recommendations for people with higher body weight than the reference value of 70 kg. However, with the increasing prevalence of both overweight and obesity reaching pandemic proportions, it appears appropriate from a consistent health perspective to recommend an additional intake of vitamin C with increased weight. Estimations based on controlled dose versus concentration studies as well as pharmacokinetic evaluations (65) suggest that an additional 10 mg/day of vitamin C should be added for each 10 kg weight gain from 70 to 100 kg in order to maintain a similar plasma concentration. Furthermore, NHANES data suggest an AR of 140 mg/day for heavier people (74). Thus, for a person weighing 100 kg or more, the additional RI is +30 mg/day.

### Smoking

Tobacco smoking has consistently been shown to result in lower vitamin C status. Consequently, some authorities have tried to estimate an additional requirement for smokers based on increased metabolic loss of vitamin C or the increased intake necessary to compensate for the difference observed between smokers and non-smokers. This has resulted in the recommendations of an additional intake of 20–80 mg/day in several European countries and the United States. As factors other than increased metabolic loss are known to contribute to the lower vitamin C status observed in smokers, it was decided to base the additional AR for smokers solely on calculations on increased vitamin C turnover. Based on radiolabelled tracer studies in smokers and non-smokers (83, 108), respectively, an additional AR was suggested to be +35 mg/day. Assuming a CV of 10%, this results in a suggested additional RI for smokers of +40 mg/day.

### Data gaps for future research

A severe limitation in the estimations of ARs for vitamin C is the limited availability of dose-response data from controlled studies with solid clinical endpoints. However, such studies are extremely difficult and expensive to conduct, and reliable biomarkers of response, as an alternative approach, have so far not been identified. Such data, if available, would provide a more thorough scientific background for choosing a relevant target plasma concentration. While RCTs constitute the best available tool to establish causality when looking at efficacy of drugs, these may have serious limitations when applied to micronutrients because of the inherent problems in establishing an appropriate and indeed relevant control group. As such, while many RCTs have been conducted, these largely suffer from various design problems, for example, studying the effects of supplementation in individuals already ingesting adequate amounts of vitamin C and in many cases also concurrent supplements in the placebo group. Finally, the often relatively short intervention period severely limits the ability of controlled trials to predict lifelong effects of vitamin C status. This increases the dependency on prospective studies in which bias and confounding is very difficult if not impossible to avoid. Indeed, multiple possible confounders have been identified for vitamin C including, for example, smoking, fruit and vegetable intake, body weight, sex, along with several other demographic, dietary, lifestyle, and anthropometric variables. One major difficulty is that the relationships between the confounders and the vitamin C intake/plasma vitamin C are relatively linear for some but not for others, making proper adjustments complicated. Also, the difficulties in translating food frequency questionnaire information into the appropriately corresponding vitamin C intake equivalent have been documented, suggesting that studies reporting measured fasted plasma concentrations are far more reliable when attempting to establish a dose-response relationship (12).

In relation to disease endpoints, it has become clear from the multitude of studies demonstrating low vitamin C concentrations in sick compared to healthy individuals that the dose versus concentration relationship changes significantly with chronic disease, suggesting that subclinical disease may also constitute a major potential confounder. Well-designed RCTs with a focus on people with low dietary intake and the correspondingly low baseline plasma concentrations of vitamin C and also investigations into the potential for supplementation to improve health for people with high risk of vitamin C deficiency would provide a better rationale for setting ARs. However, as mentioned earlier, maintaining already good health requires considerably less vitamin C per day than achieving normalization of vitamin C status for an already sick individual. Thus, the predefined research question needs to be better integrated into the design and dose selection, where, for example, establishing the dose versus concentration relationship for healthy individuals in various subpopulations presumably requires relatively small doses in the 50–200 mg/day range and, for example, preventing disease (progression) in high-risk individuals with poor vitamin C status may require significantly higher doses in the 500–2,000 mg/day range. Moreover, detailed dose-concentration estimates for various subpopulations would also provide an opportunity to validate the isometric scaling approach used for estimating RIs for women, children, and adolescents.

## Conflict of interest and funding

The authors have not received any funding or benefits from industry or elsewhere to conduct this study.
